# An n-type Schottky contact with low tunneling resistance in a 2D FeB_2_/SiC heterostructure

**DOI:** 10.1039/d6na00108d

**Published:** 2026-04-29

**Authors:** Nguyen Q. Cuong, Le Phuong Long, Huynh V. Phuc, Nguyen D. Hien

**Affiliations:** a Institute of Research and Development, Duy Tan University Da Nang 550000 Vietnam nguyenquangcuong3@duytan.edu.vn; b School of Engineering & Technology, Duy Tan University Da Nang 550000 Vietnam; c Center of Scientific Research and Application, Lac Hong University No. 10 Huynh Van Nghe Str, Tran Bien Ward Dong Nai Province Vietnam phuonglong@lhu.edu.vn; d Division of Physics, School of Education, Dong Thap University Dong Thap 870000 Vietnam hvphuc@dthu.edu.vn; e Nha Trang Center Ethnic Minority Pre-University School No. 46 Nguyen Thien Thuat Str., Nha Trang Ward Khanh Hoa Province Vietnam

## Abstract

The design of low-resistance metal–semiconductor contacts remains a critical challenge for the development of high-performance two-dimensional (2D) electronic devices. In this work, we design and systematically investigate the structural stability, electronic properties, and interfacial contact characteristics of a 2D FeB_2_/SiC metal–semiconductor heterostructure using first-principles calculations. The FeB_2_/SiC heterostructure is found to be energetically stable with weak van der Waals (vdW) interlayer interaction, thereby preserving the intrinsic electronic properties of the individual monolayers. Owing to the larger work function of FeB_2_ compared to that of SiC, charge transfer occurs from the SiC layer to the FeB_2_ layer, resulting in interfacial charge redistribution and downward band bending in the SiC layer. Consequently, an n-type Schottky contact is formed at the interface with a Schottky barrier height of about 0.70 eV. Projected density-of-states analysis indicates negligible metal-induced gap states (MIGS) at the interface, implying weak Fermi-level pinning. Moreover, the FeB_2_/SiC heterostructure exhibits a low tunneling resistance of 1.40 × 10^−9^ Ω cm^2^, confirming the formation of a low-resistance contact. These results demonstrate that the FeB_2_/SiC heterostructure is a promising two-dimensional metal–semiconductor contact for high-performance and low-power electronic devices.

## Abstract 2

The design of low-resistance metal–semiconductor contacts remains a critical challenge for the development of high-performance two-dimensional (2D) electronic devices.

## Graphical abstract

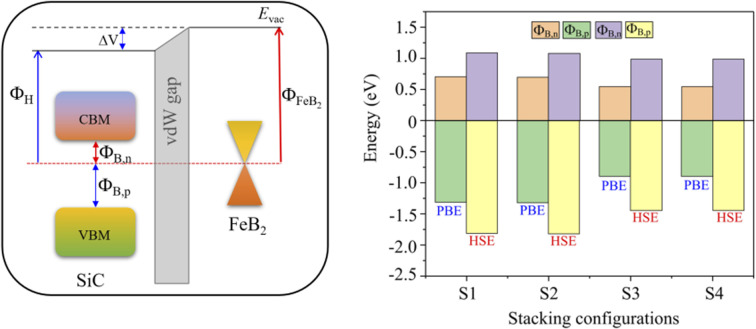

## 1 Introduction

The rapid progress of nanoelectronics and next-generation electronic devices has stimulated extensive research on two-dimensional (2D) materials^1–3^ and their van der Waals (vdW) heterostructures.^4–6^ By vertically stacking atomically thin layers with distinct electronic characteristics, vdW heterostructures enable unprecedented flexibility in materials integration without the constraint of lattice matching.^7–9^ This unique feature allows for precise tuning of electronic, optical, and transport properties at the atomic scale, making 2D heterostructures promising building blocks for high-performance electronic and optoelectronic applications.^10–14^ For example, 2D metallic MBenes have been demonstrated to serve as promising electrodes, enabling the formation of p-type ohmic contacts when integrated with MoS_2_ semiconductors.^13,14^

In semiconductor-based devices, the nature of the metal–semiconductor contact plays a decisive role in determining carrier injection efficiency and overall device performance.^15^ For 2D semiconductors, contact characteristics are strongly governed by interfacial phenomena, including Fermi level pinning, interfacial charge transfer, and interface-induced dipoles.^16–18^ These effects frequently result in the formation of Schottky barriers, which can severely hinder carrier transport and lead to increased contact resistance. Consequently, identifying suitable 2D metallic electrodes capable of forming low-barrier Schottky contacts or ideally ohmic contacts with 2D semiconductors remains a critical challenge in the development of high-performance 2D electronic devices.^19–24^

2D silicon carbide (SiC) nanosheets have emerged as promising 2D materials owing to their outstanding physical properties, including a wide band gap, high saturated carrier mobility, excellent thermal conductivity, and environmental benignity.^25–27^ Notably, monolayer 2D SiC was first theoretically predicted based on first-principles calculations,^26^ and has subsequently been experimentally realized *via* bottom-up epitaxial growth methods.^28^ Furthermore, compared to bulk wurtzite or zinc blende SiC,^29^ the 2D planar SiC possesses a dangling-bond-free surface and is particularly suitable for forming van der Waals (vdW) heterostructures, thereby avoiding lattice mismatch constraints typically encountered in conventional bulk heterostructures. Owing to these advantageous characteristics, 2D SiC represents a highly promising semiconducting platform for integration with metallic materials, facilitating the realization of metal–semiconductor heterostructures and efficient electrical contacts in next-generation 2D electronic devices. Previously, Huang *et al.*^30^ systematically investigated the interfacial properties of conventional bulk metals, such as Ag, Al, Ni, Pt, and Ti, in contact with a SiC monolayer using first-principles calculations. Their results revealed that these 3D metals generally form strongly chemisorbed interfaces with SiC, leading to Fermi-level pinning effects and metal-induced gap states (MIGS). In addition, the strong interaction at the interface may induce structural distortion or degrade the intrinsic electronic characteristics of the SiC layer. In contrast, 2D metallic materials provide an alternative route for contact engineering. Owing to their atomically thin nature, 2D metals can form vdW interfaces with SiC semiconductors, which helps preserve the intrinsic electronic structure, suppress metal-induced gap states, and enable more flexible tuning of the Schottky barrier. To date, numerous experimental and theoretical efforts have been devoted to the fabrication and characterization of ohmic contacts to the 2D SiC material, achieving extremely low specific contact resistance and excellent thermal and chemical stability.^31,32^ For instance, Si *et al.*^31^ predicted that 2D SiC, when integrated with a 2D metallic MoSH monolayer, can form a p-type quasi-ohmic contact, highlighting the potential of 2D SiC for contact engineering in metal–semiconductor vdW heterostructures.

Recently, by introducing commonly utilized Fe atoms into a 2D honeycomb boron network, a novel FeB_2_ monolayer has been theoretically designed and predicted to be a Dirac material, exhibiting linear band dispersion near the Fermi level and a Fermi velocity on the same order of magnitude as that of graphene.^33^ It has been demonstrated that the FeB_2_ monolayer is dynamically, mechanically and thermodynamically stable,^34,35^ and displays pronounced metallic characteristics with a high density of states at the Fermi level and a high carrier mobility of 5.8 × 10^4^ cm^2^ V^−1^ s^−1^.^36^ These features suggest that FeB_2_ is a promising candidate for use as a 2D metallic electrode in nanoelectronic applications. Despite these promising properties, the potential of FeB_2_ in vdW heterostructures, particularly for contact engineering applications, has not yet been systematically explored. In particular, it remains unclear how its Dirac metallic nature influences key interfacial phenomena such as charge transfer, band alignment, and Schottky barrier formation when coupled with 2D semiconductors. A detailed investigation of these aspects is therefore essential to determine whether FeB_2_ can offer distinct advantages over conventional metallic contacts and to reveal the underlying physical mechanisms governing contact behavior in Dirac metal–semiconductor systems.

Therefore, a comprehensive investigation of the FeB_2_/SiC 2D metal–semiconductor heterostructure is highly desirable to elucidate the interfacial electronic properties, band alignment, and contact behavior. Such a study is expected to provide fundamental insights into metal–semiconductor contacts in the 2D limit and to assess the feasibility of employing FeB_2_ as a low-resistance electrode for SiC-based 2D electronic and optoelectronic devices.

## 2 Computational details

All first-principles calculations in this work were performed within the framework of density functional theory (DFT), as implemented in the Quantum Espresso (PWscf).^37,38^ The interaction between the valence electrons and ionic cores was described using the projector augmented-wave (PAW) method.^39^ The electronic exchange–correlation energy was treated using the generalized gradient approximation (GGA) with the Perdew–Burke–Ernzerhof (PBE) functional.^40^ A plane-wave basis set with a kinetic energy cutoff of 510 eV was employed to ensure good convergence of total energies and electronic structures. The Brillouin zone was sampled using a Monkhorst–Pack *k*-point mesh of 15 × 15 × 1 for geometry optimization and electronic structure calculations. All atomic positions were fully relaxed until the residual forces on each atom were less than 0.01 eV Å^−1^, and the total energy convergence criterion was set to 10^−6^ eV. To eliminate spurious interactions between periodic images along the out-of-plane direction, a vacuum layer of 30 Å was introduced perpendicular to the 2D plane. The weak vdW interactions between the FeB_2_ and SiC layers were taken into account using the DFT-D3 method of Grimme.^41^

## 3 Results and discussion

**Fig. 1:**
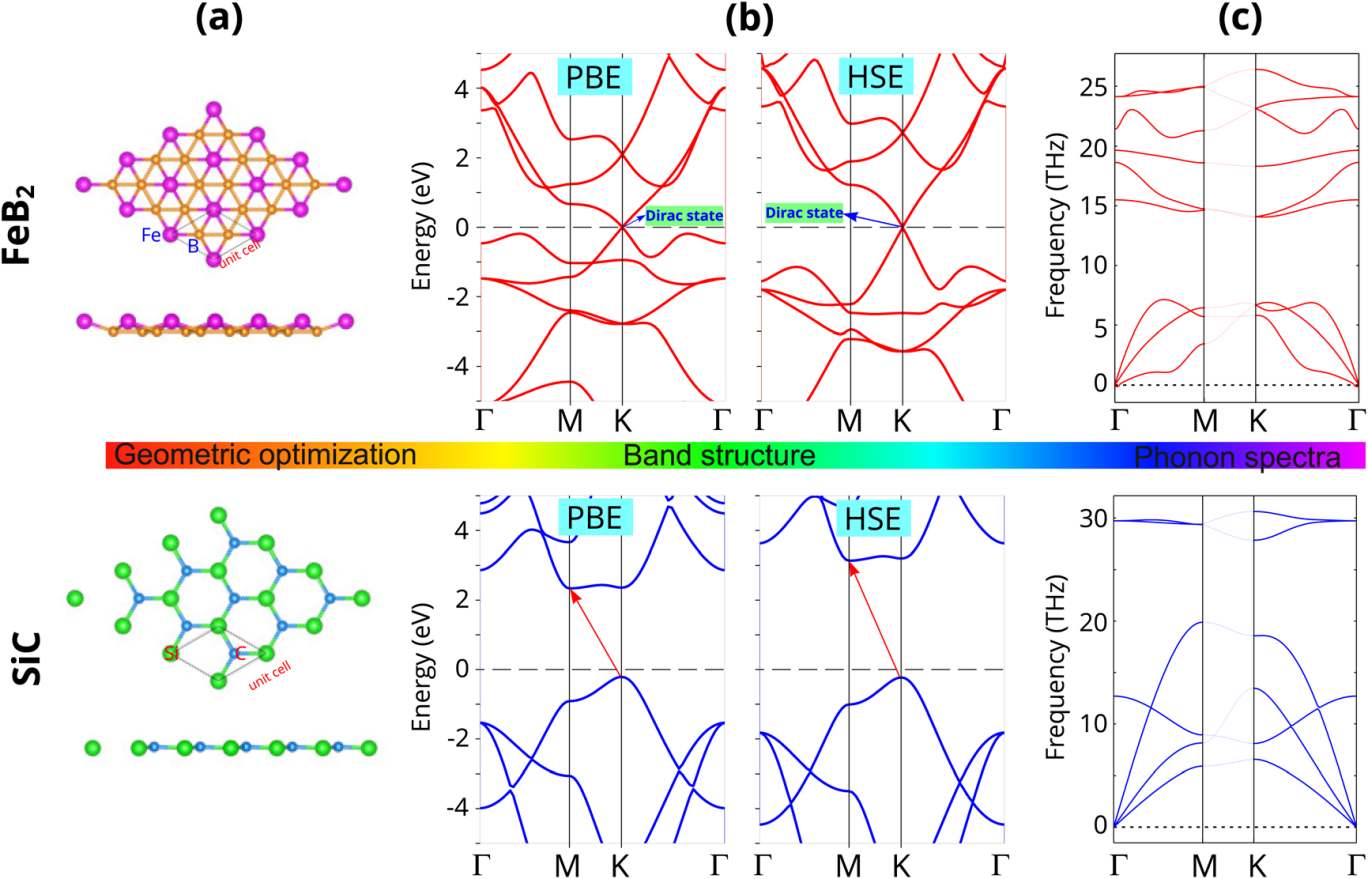
(a) Optimized atomic structures (b) band structures and (c) phonon spectra of the constituent Fe_2_B and SiC monolayers. Purple and orange balls represent the Fe and B atoms, respectively, while green and blue balls indicate the Si and C atoms, respectively.

We first investigate the intrinsic properties of the constituent FeB_2_ and SiC monolayers by analyzing their atomic structures and electronic characteristics. The optimized atomic structures of the FeB_2_ and SiC monolayers are shown in Fig. 1(a). The FeB_2_ monolayer crystallizes in the hexagonal *P*6/3*m* space group, featuring a honeycomb boron network with Fe atoms embedded at the centers of six-membered boron rings. Consequently, the FeB_2_ monolayer exhibits a quasi-planar geometry. Each Fe atom is positioned at a vertical distance of 0.63 Å above the hexagonal plane, which is in good agreement with previous reports.^33,42^ The primitive unit cell of the FeB_2_ monolayer contains two B atoms and one Fe atom. In addition, the lattice parameter of the FeB_2_ monolayer is predicted to be *a* = *b* = 3.18 Å, which is very close to the previously reported value of *a* = *b* = 3.177 Å^33^. Similarly, the SiC monolayer exhibits a planar hexagonal honeycomb structure, with all constituent atoms lying in the same plane. The optimized lattice parameters of the SiC monolayer are *a* = *b* = 3.10 Å, which are in good agreement with the experimental value (*a* = *b* = 3.08 Å)^43^ and previous theoretical studies.^44^

The electronic band structures of the FeB_2_ and SiC monolayers are presented in Fig. 1(b). The FeB_2_ monolayer exhibits a Dirac cone with linear band dispersion near the Fermi level at the *K* point, analogous to that observed in graphene,^45^ indicating its Dirac metallic character. In contrast, the SiC monolayer is a semiconductor with an indirect band gap. The valence band maximum is located at the *K* point, while the conduction band minimum occurs at the *M* point. The calculated band gaps of the SiC monolayer are 2.55 eV and 3.37 eV at the PBE and HSE levels, respectively, which are consistent with previous theoretical results.^44,46^ Importantly, despite quantitative differences in the band-gap values, both the conventional PBE and hybrid HSE functionals reliably reproduce the intrinsic electronic characteristics of FeB_2_ and SiC, validating their use for the subsequent electronic and interfacial property analyses.

To further assess the dynamical stability of the constituent monolayers, we analyze their phonon spectra, as presented in Fig. 1(c). For both FeB_2_ and SiC monolayers, no imaginary phonon modes are observed throughout the entire Brillouin zone, indicating their dynamical stability. The phonon spectrum of the FeB_2_ monolayer exhibits well-defined acoustic and optical branches, with the acoustic modes displaying the expected linear dispersion near the *Γ* point. In contrast, the SiC monolayer shows a wider phonon frequency range. These results confirm that both monolayers are dynamically stable and can serve as reliable building blocks for constructing vdW heterostructures.

**Fig. 2:**
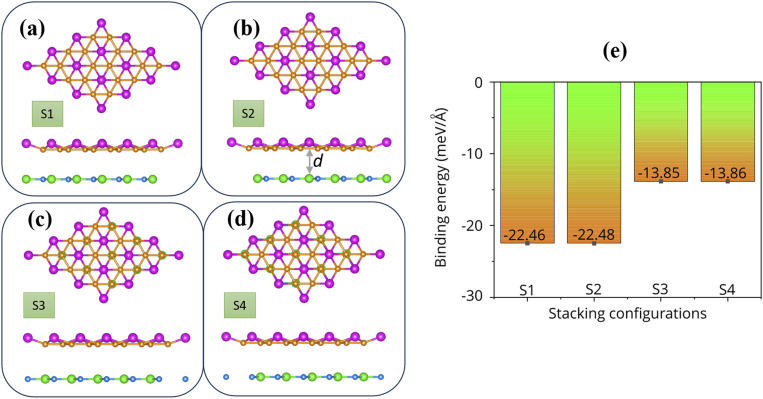
Top and side views of the optimized atomic structures of the FeB_2_/SiC heterostructure for different stacking configurations: (a) S1, (b) S2, (c) S3, and (d) S4. Fe and B atoms are represented by purple and orange spheres, respectively, while Si and C atoms are shown in green and blue, respectively. (e) Calculated binding energies corresponding to the four stacking configurations.

We now construct the FeB_2_/SiC heterostructure by vertically stacking the metallic FeB_2_ monolayer on top of the semiconducting SiC monolayer. The FeB_2_/SiC heterostructure is built by placing a unit cell of the FeB_2_ layer onto a (1 × 1) unit cell of the SiC layer. The optimized lattice constant of the heterostructure is 3.14 Å, which is taken as the average of the lattice constants of FeB_2_ (3.18 Å) and SiC (3.10 Å). This lattice matching induces a slight compressive strain in the FeB_2_ layer, while the SiC layer experiences a small tensile strain. The lattice mismatch in the heterostructure is estimated to be approximately 2%, which is sufficiently small to ensure structural stability and to minimize strain-induced perturbations to the electronic properties. Four representative high-symmetry stacking configurations, denoted as S1, S2, S3, and S4, are considered, as illustrated in Fig. 2. These stacking configurations are selected based on high-symmetry atomic registries between the two monolayers, corresponding to different relative positions of Fe and B atoms with respect to the Si and C sublattices. After full structural relaxation, the equilibrium interlayer spacing *d* between the FeB_2_ and SiC layers is found to be 3.18, 3.17, 3.58, and 3.58 Å for the S1, S2, S3, and S4 stacking configurations, respectively. Notably, the interlayer distances in the S1 and S2 configurations are smaller than those in S3 and S4, indicating relatively stronger interlayer interactions. This difference can be attributed to the distinct atomic registries at the interface: in the S1 and S2 configurations, Fe atoms are positioned directly above Si atoms, whereas in the S3 and S4 configurations, Fe atoms are located above C atoms. Consequently, the stronger interaction between Fe and Si atoms associated with the larger atomic radius of Si compared to C leads to a reduced equilibrium interlayer spacing in the S1 and S2 stacking configurations. Moreover, the obtained interlayer spacings *d* are consistent with those of previously reported metal/semiconductor vdW heterostructures, such as MoSSe/SiC,^47^ ZnO/GaN,^48^ germanene/SiC,^49^ MoS_2_/SiC^50^ and C_3_N/phosphorene,^51^ where the spacings typically range from 2.5 to 3.6 Å, further confirming the vdW nature of the interfacial interaction.

To quantitatively evaluate the interfacial stability of the FeB_2_/SiC heterostructure, we calculate the binding energy for all considered stacking configurations. The binding energy *E*_b_ is defined as follows:1
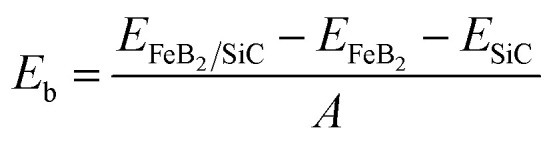
where *E*_FeB_2_/SiC_ is the total energy of the heterostructure and *E*_FeB_2__ and *E*_SiC_ are the total energies of the isolated FeB_2_ and SiC monolayers in their optimized geometries, respectively. Here, *A* denotes the interfacial area of the heterostructure. The calculated binding energies for the FeB_2_/SiC heterostructure under different stacking configurations are summarized in Fig. 2(e). A negative binding energy indicates that the formation of the FeB_2_/SiC heterostructure is energetically favorable, confirming its thermodynamic stability. The relatively small magnitude of *E*_b_ suggests that the interaction between the FeB_2_ and SiC layers is dominated by weak vdW forces rather than strong covalent or ionic bonding. This vdW-type interlayer coupling preserves the intrinsic electronic properties of the individual monolayers, which is beneficial for constructing high-quality heterostructures without introducing significant lattice distortion or chemical hybridization. Among all the considered stacking configurations, the S2 stacking is identified as the most energetically favorable, as it exhibits the most negative binding energy together with the shortest equilibrium interlayer distance.

**Fig. 3:**
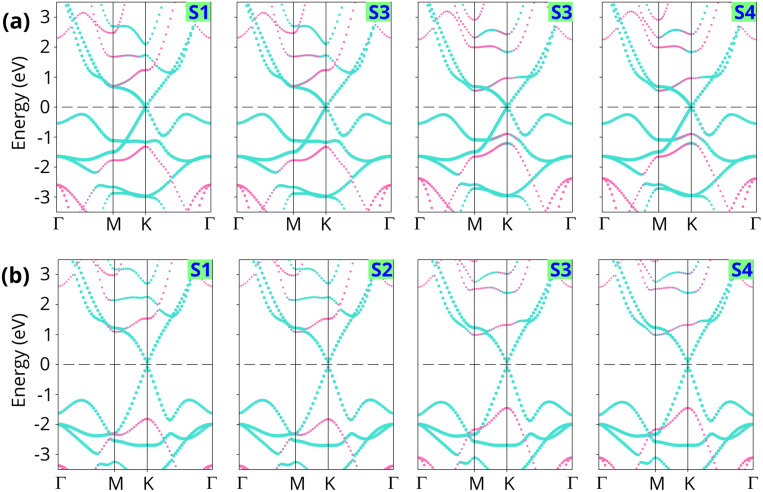
Projected band structures of the FeB_2_/SiC heterostructure for different stacking configurations obtained by the (a) PBE and (b) HSE functionals. Blue and purple lines represent the band projections of the FeB_2_ and SiC layers, respectively. The Fermi level is set to zero.

**Fig. 4:**
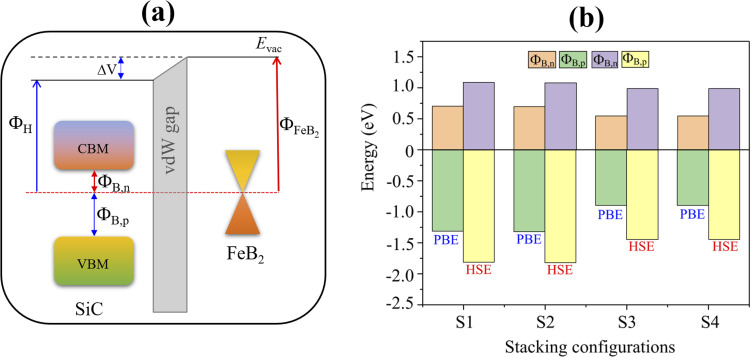
(a) Schematic illustration of the formation of the Schottky contact with Schottky barriers and (b) calculated Schottky barriers at the interface of the FeB_2_/SiC metal–semiconductor heterostructure using the PBE and HSE functionals.

Next, we analyze the electronic band structure of the FeB_2_/SiC heterostructure to elucidate its interfacial electronic properties, as illustrated in Fig. 3. The results indicate that the intrinsic electronic characteristics of the constituent FeB_2_ and SiC monolayers are largely preserved upon heterostructure formation. In particular, the FeB_2_ monolayer retains its Dirac cone with linear band dispersion near the Fermi level at the *K* point, confirming its Dirac metallic nature. Meanwhile, the SiC monolayer continues to exhibit an indirect semiconducting character, with the valence band maximum and conduction band minimum located at the *K* and *M* points, respectively. This preservation of the intrinsic band features can be attributed to the weak vdW interlayer coupling between FeB_2_ and SiC. Furthermore, the combination of the FeB_2_ and SiC monolayers leads to a decrease in the band gap of the SiC semiconductor as compared to the pristine one. More interestingly, due to the contact between metallic FeB_2_ and semiconducting SiC, the FeB_2_/SiC system naturally forms a metal–semiconductor heterostructure, in which the nature of the interfacial contact is governed by band alignment and charge redistribution. As a consequence, a Schottky contact is formed at the FeB_2_/SiC interface, as shown in Fig. 4(a). The Schottky barrier height is determined by the relative position of the Fermi level (*E*_F_) of FeB_2_ with respect to the conduction band minimum (*E*_CBM_) and valence band maximum (*E*_VBM_) of the SiC layer as follows:

2 *Φ*_B,n_ = *E*_CBM_ − *E*_F_

and

3 *Φ*_B,p_ = *E*_F_ − *E*_CBM_

The calculated Schottky barriers at the interface of the metal–semiconductor FeB_2_/SiC heterostructure are depicted in Fig. 4(b). The Schottky barrier heights for electrons (*Φ*_B,n_) and holes (*Φ*_B,p_) are calculated to be 0.71 and 1.31 eV for the S1 stacking configuration, 0.70 and 1.32 eV for S2, 0.55 and 0.89 eV for S3, and 0.54 and 0.89 eV for S4, respectively. It is clearly observed that the electron Schottky barrier *Φ*_B,n_ is consistently smaller than the hole barrier *Φ*_B,p_ for all stacking configurations. This behavior indicates that the FeB_2_/SiC heterostructure forms an n-type Schottky contact irrespective of the stacking arrangement. One can find that the observed difference in SBH values among the stacking configurations originates from the variation in interfacial interaction and charge redistribution between the FeB_2_ and SiC layers. Moreover, these Schottky barrier values are lower than those reported for other representative 2D metal/semiconductor heterostructures, such as graphene/Mo(W)Si_2_N_4_ (0.98 (0.78 eV))^52^ and MXene/MoSi_2_N_4_ (0.97 eV).^53^ To further validate the reliability of the results, the Schottky barriers are also evaluated using the HSE hybrid functional, which provides a more accurate description of the band gap of the SiC layer. As expected, the HSE functional yields larger absolute Schottky barrier heights compared to PBE. Specifically, the calculated *Φ*_B,n_ and *Φ*_B,p_ values are 1.09 and 1.81 eV for S1, 1.08 and 1.82 eV for S2, 0.99 and 1.44 eV for S3, and 0.98 and 1.44 eV for S4, respectively. Notably, despite the quantitative increase in barrier heights, the n-type Schottky contact nature remains unchanged, demonstrating that the contact characteristics are robust against the choice of the exchange–correlation functional.

**Fig. 5:**
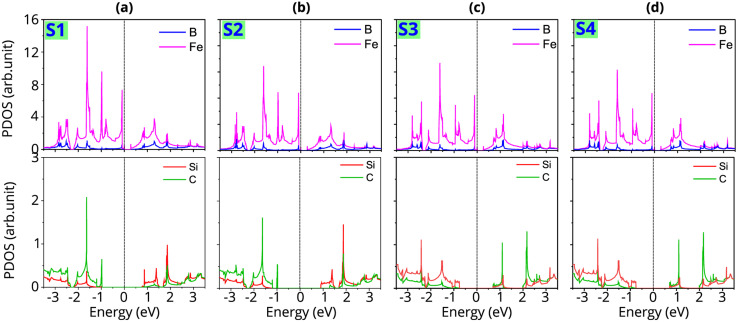
Projected density of states (PDOS) of all atoms in the metal–semiconductor FeB_2_/SiC heterostructure for the different stacking configurations of (a) S1, (b) S2, (c) S3 and (d) S4. The Fermi level is set to zero.

To gain deeper insight into the physical origin of the electronic properties and the formation of the Schottky contact in the FeB_2_/SiC heterostructure, we further analyze the projected density of states (PDOS), as shown in Fig. 5. The PDOS results reveal that the electronic states near the Fermi level are predominantly contributed by the Fe-d orbitals of the FeB_2_ layer, confirming its metallic character. In contrast, the SiC layer exhibits a clear band gap around the Fermi level, with the valence band mainly composed of C-p states and the conduction band dominated by Si-p states. Importantly, no significant orbital hybridization between the electronic states of FeB_2_ and SiC is observed near the Fermi level, indicating weak interlayer coupling at the interface. Moreover, the absence of pronounced interface-induced mid-gap states in the PDOS suggests that Fermi-level pinning is effectively suppressed, allowing the Schottky barrier height to be primarily governed by the intrinsic band alignment between the FeB_2_ and SiC layers rather than by defect-related interface states. Furthermore, it should be noted that in metal–semiconductor heterojunctions, ohmic contacts are generally preferred for minimizing contact resistance, whereas the presence of a Schottky barrier in Schottky contacts may impede carrier injection. However, Schottky contacts also play a crucial role in functional devices such as rectifiers, sensors, and photodetectors, where controlled carrier transport is required. In the vdW FeB_2_/SiC heterostructure, the formation of a Schottky contact originates from weak interfacial interactions, which helps preserve the intrinsic electronic properties of the SiC layer and suppress metal-induced gap states. Moreover, the Schottky barrier enables effective regulation of carrier injection and leakage current, which is particularly important for low-power device applications.

**Fig. 6:**
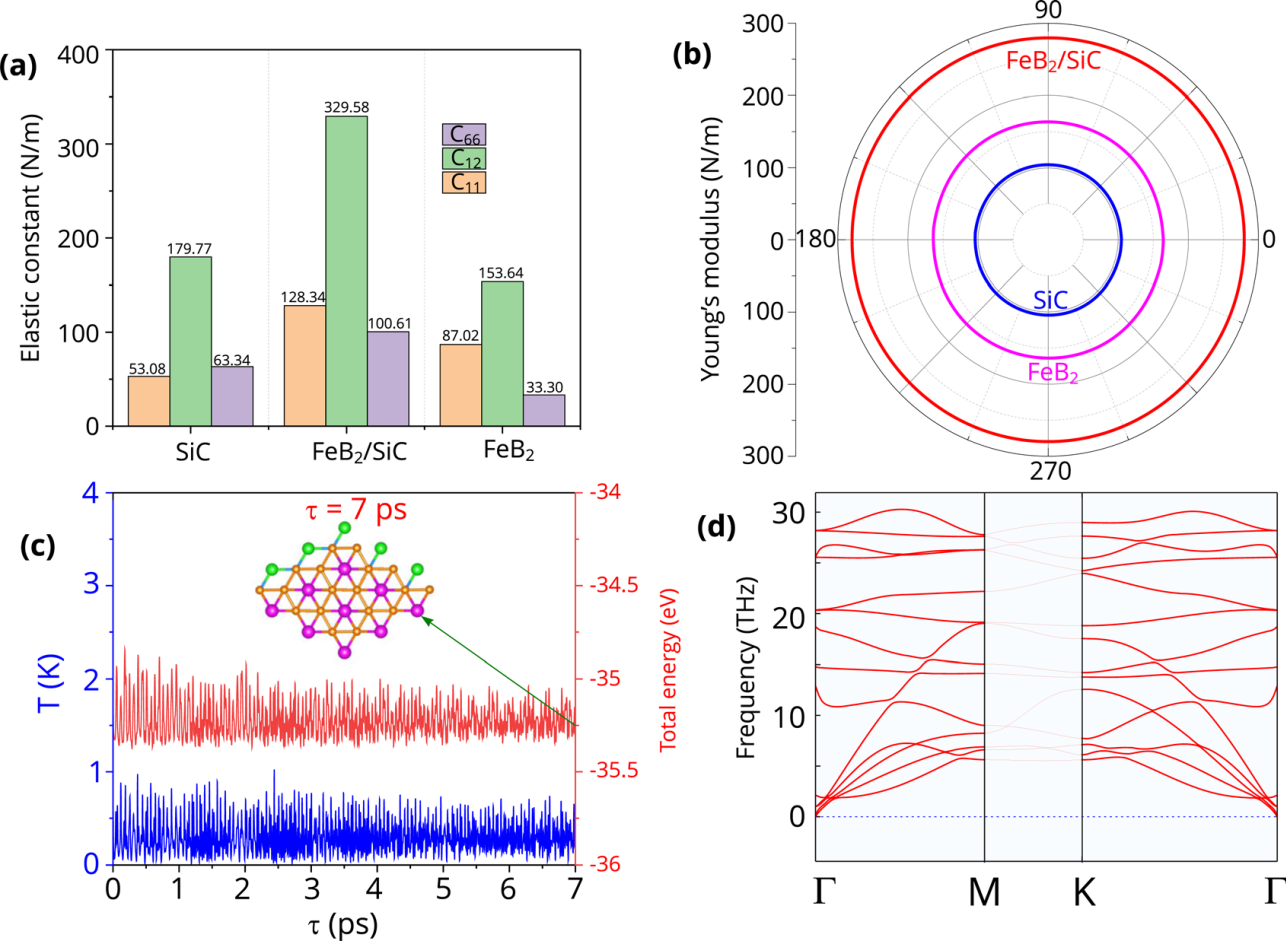
(a) Elastic constants, (b) Young's modulus, (c) AIMD simulations of the total energy and temperature as a function of simulation time and (d) phonon spectra of the FeB_2_/SiC heterostructure.

We now further evaluate the stability of the FeB_2_/SiC heterostructure by evaluating the mechanical properties, AIMD simulation and phonon spectra. The mechanical stability of the most favorable S2-stacked FeB_2_/SiC heterostructure is systematically evaluated by calculating its elastic constants and Young's modulus. The elastic constants *C*_*ij*_ are obtained using the strain–energy method within the harmonic elastic regime. In this approach, a series of small in-plane strains are applied to the fully relaxed structure, and the resulting total energies are fitted to the quadratic strain–energy relationship:^54^4
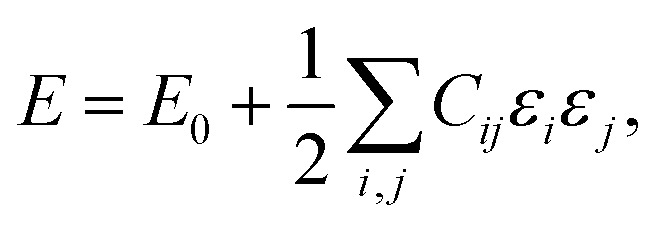
where *E*_0_ is the total energy of the equilibrium structure, *C*_*ij*_ are the elastic constants, and *ε*_*i*_ and *ε*_*j*_ denote the strain components. For a 2D hexagonal lattice, the in-plane elastic behavior is characterized by two independent elastic constants, *C*_11_ and *C*_12_, with the symmetry relations *C*_11_ = *C*_22_ and *C*_66_ = (*C*_11_ − *C*_12_)/2. The calculated elastic constants of the FeB_2_/SiC heterostructure, together with those of the constituent FeB_2_ and SiC monolayers, are presented in Fig. 6(a). The elastic constants *C*_11_, *C*_12_, and *C*_66_ of the FeB_2_/SiC heterostructure are obtained to be 128.34, 329.58, and 100.61 N m^−1^, respectively. These elastic constants satisfy the Born–Huang stability criteria for 2D hexagonal crystals, namely *C*_11_ > 0 and *C*_11_ − *C*_12_ > 0, confirming that the FeB_2_/SiC heterostructure is mechanically stable. In addition, the elastic constants *C*_*ij*_ of the FeB_2_/SiC heterostructure are larger than those of the individual FeB_2_ and SiC monolayers, indicating an enhanced in-plane stiffness upon heterostructure formation. This improvement can be attributed to the synergistic mechanical coupling between the two constituent layers, which effectively reinforces the structural rigidity while maintaining the vdW nature of the interlayer interaction.

We further evaluate the in-plane Young's modulus to gain deeper insight into the directional mechanical stiffness of the FeB_2_/SiC heterostructure. Based on the calculated elastic constants, the angle-dependent Young's modulus *Y*(*θ*) for a 2D hexagonal system can be expressed as follows:5
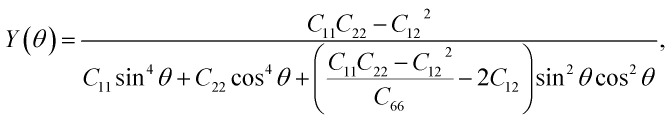


As shown in Fig. 6(b), the Young's modulus of the FeB_2_/SiC heterostructure exhibits circular symmetry, confirming its mechanical isotropy in the basal plane. The calculated Young's modulus of the FeB_2_/SiC heterostructure is 279.60 N m^−1^, which is significantly larger than those of the constituent FeB_2_ (104.34 N m^−1^) and SiC (164.10 N m^−1^) monolayers. This enhancement indicates that the formation of the heterostructure leads to improved in-plane stiffness, arising from the synergistic mechanical coupling between the two layers. Such enhanced mechanical rigidity is beneficial for maintaining structural stability under external strain, further supporting the robustness of the FeB_2_/SiC heterostructure for practical applications in flexible and high-performance nanoelectronic devices.

To further assess the stability of the FeB_2_/SiC heterostructure beyond the mechanical response, we next investigate its thermal stability using *ab initio* molecular dynamics (AIMD) simulations, as shown in Fig. 6(c). The AIMD simulations are performed at an elevated temperature to examine the structural robustness of the system under thermal fluctuations. As illustrated in Fig. 6(c), the total energy of the FeB_2_/SiC heterostructure exhibits only small oscillations around an equilibrium value throughout the simulation, while the temperature remains well controlled without abrupt deviations. Importantly, no noticeable structural reconstruction, bond breaking, or layer separation is observed during the simulation process, indicating that the heterostructure retains its structural integrity under thermal perturbations. These results demonstrate that the FeB_2_/SiC heterostructure possesses excellent thermal stability, further confirming its feasibility for experimental realization and reliable operation in practical device environments. Furthermore, the phonon spectrum of the FeB_2_/SiC heterostructure exhibits no imaginary phonon modes throughout the entire Brillouin zone, indicating that the structure is dynamically stable against lattice vibrations. The absence of soft modes near the *Γ* point further confirms that the heterostructure corresponds to a true minimum on the potential energy surface.

**Fig. 7:**
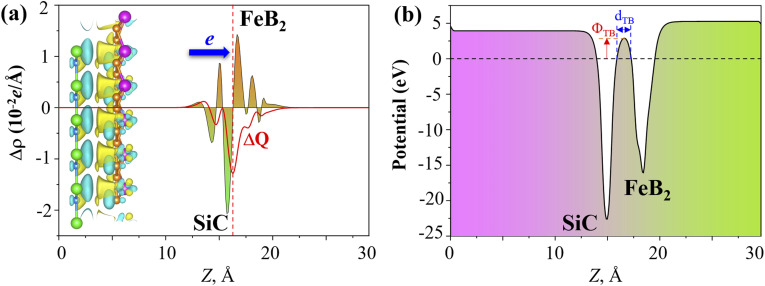
(a) Planar-averaged charge density difference Δ*ρ*(*z*) and total charge transfer Δ*Q* of the FeB_2_/SiC heterostructure. The inset shows the 3D charge density difference isosurface, where yellow and cyan regions represent electron accumulation and electron depletion, respectively. (b) Plane-averaged electrostatic potential along the out-of-plane direction for the FeB_2_/SiC heterostructure.

To further elucidate the interfacial charge transfer of the FeB_2_/SiC heterostructure, the planar-averaged charge density difference Δ*ρ*(*z*), the total amount of charge transfer Δ*Q*, and the electrostatic potential are presented in Fig. 7(a). The charge density difference is defined as follows:

6 Δ*ρ*(**r**) = *ρ*_FeB_2_/SiC_(**r**) − *ρ*_FeB_2__(**r**) − *ρ*_SiC_(**r**)

where *ρ*_FeB_2_/SiC_(**r**) is the total charge density of the heterostructure, and *ρ*_FeB_2__(**r**) and *ρ*_SiC_(**r**) represent the charge densities of the isolated FeB_2_ and SiC monolayers, respectively. The total amount of charge transfer Δ*Q* across the interface is then evaluated by integrating the planar-averaged charge density difference along the out-of-plane direction as follows:7
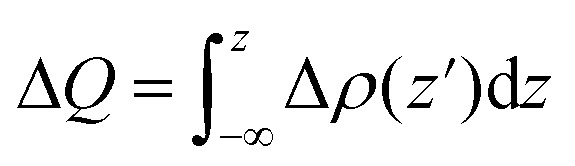


As illustrated in Fig. 7(a), charge accumulation is predominantly observed on the FeB_2_ side of the interface, whereas charge depletion mainly occurs on the SiC layer. Consistently, the planar-averaged charge density difference shows positive values of Δ*ρ*(*z*) on the FeB_2_ side and negative values on the SiC side. According to the adopted convention, positive and negative Δ*ρ*(*z*) correspond to electron accumulation and electron depletion, respectively. This observation clearly indicates that electrons are transferred from the SiC monolayer to the FeB_2_ monolayer upon heterostructure formation. In addition, by analyzing the obtained total charge transfer Δ*Q*, it is found that Δ*Q* < 0, further confirming that the SiC layer experiences electron depletion while the FeB_2_ layer gains electrons. Consequently, the SiC monolayer acts as an electron donor, while the FeB_2_ monolayer serves as an electron acceptor at the interface. This donor–acceptor charge transfer leads to the formation of an interfacial dipole, which modifies the electrostatic potential profile and induces band bending in the SiC layer near the interface. The resulting interfacial dipole and band bending reduce the electron Schottky barrier relative to the hole barrier, thereby promoting the formation of an n-type Schottky contact in the FeB_2_/SiC heterostructure.

Furthermore, the electrostatic potential profile of the FeB_2_/SiC heterostructure is plotted in Fig. 7(b) to gain deeper insight into the charge injection efficiency. Owing to the larger work function of FeB_2_ compared to that of SiC, electrons tend to transfer from the SiC layer to the FeB_2_ layer upon contact, leading to charge redistribution at the interface when the two materials are stacked. In addition, from the planar-averaged electrostatic potential, a noticeable potential drop across the interface can be observed, indicating the formation of an interfacial built-in electric field. This built-in electric field facilitates electron injection from SiC into FeB_2_, which is beneficial for efficient charge separation and transport. Such favorable band alignment and interfacial charge transfer suggest that the FeB_2_/SiC heterostructure is promising for electronic and optoelectronic device applications.

To evaluate the quality of the interfacial electrical contact and to quantitatively assess carrier transport across the FeB_2_/SiC interface, it is essential to determine the electron tunneling probability and the corresponding tunneling specific contact resistivity. These two quantities directly characterize the efficiency of charge injection through the interfacial potential barrier and serve as key figures of merit for contact performance in nanoscale electronic devices. Within the framework of a simplified one-dimensional rectangular tunneling barrier model, the electron tunneling probability *T*_TB_ can be estimated using the Wentzel–Kramers–Brillouin (WKB) approximation. In this model, the interfacial potential barrier is characterized by an effective barrier height *Φ*_TB_ and the barrier width *d*_TB_, both of which are extracted from the planar-averaged electrostatic potential profile along the out-of-plane direction. Accordingly, the tunneling probability is given as follows:8
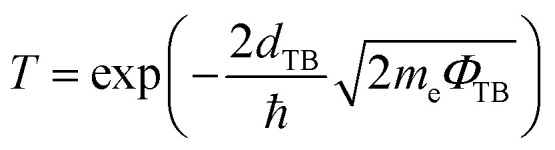


Based on the calculated tunneling probability, the tunneling specific contact resistivity *ρ*_t_ can be further evaluated as follows:9
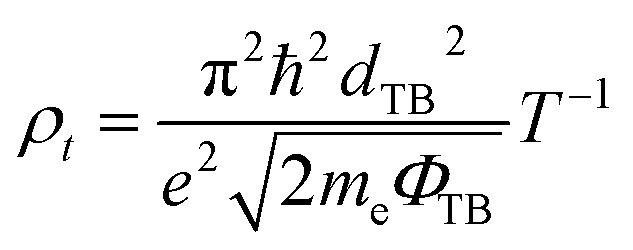


The effective tunneling barrier height *Φ*_TB_ and barrier width *d*_TB_ of the FeB_2_/SiC heterostructure are extracted from the planar-averaged electrostatic potential profile and are found to be 2.89 eV and 1.38 Å, respectively. Based on eqn (8), the tunneling probability across the vdW gap is estimated to be approximately 9%, indicating a relatively transparent interfacial barrier. The tunneling resistance is strongly dependent on the tunneling probability and the barrier width and height of the heterostructure. Furthermore, the corresponding tunneling specific contact resistivity is evaluated to be on the order of 1.4 × 10^−9^ Ω cm^2^. It is interesting to note that the obtained value of *ρ*_t_ is comparable to those reported for other ultra-low contact resistance interfaces between semimetals or metals and semiconductors, such as Bi/MoS_2_,^55^ 3D metals/MoS_2_,^56^ semimetals/TMDs,^57,58^ 3D metals/Mo(W)Si_2_N_4_,^16,59^ MBenes/MoS_2_,^13,14^ MXenes/MoSi_2_N_4_ (ref. 53 and 60) and graphene-based heterostructures.^61–63^ This comparison indicates that the FeB_2_/SiC heterostructure can form an efficient electrical contact with highly favorable charge injection characteristics, making it a promising candidate for high-performance 2D electronic devices. Moreover, the FeB_2_/SiC heterostructure is composed of earth-abundant and low-risk elements, indicating good compatibility with sustainability criteria for next-generation electronics.^64^

In order to further evaluate the contact performance of the FeB_2_/SiC heterostructure, we estimate the contact resistance based on the Landauer formalism as follows:^65,66^10
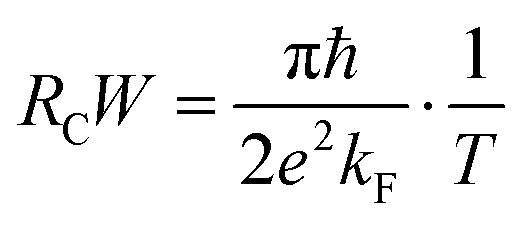
where *e* and *ℏ* are the elementary charge and reduced Planck constant, respectively, and *T* is the tunneling probability. The Fermi wave vector is defined as 
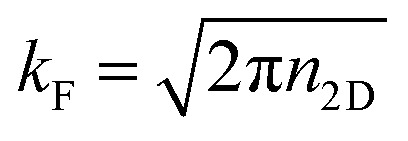
, where *n*_2D_ is the 2D carrier concentration of the semiconductor, which can be estimated from the effective mass and the relative position of the Fermi level with respect to the band edges.^13,66^ Our calculations show that the contact resistance of the FeB_2_/SiC heterostructure is as high as 2.73 × 10^3^ Ω µm, which can be attributed to the relatively large Schottky barriers. This value of the contact resistance is still higher than those reported for semimetal/semiconductor contacts^55^ and MBene/MoS_2_ heterostructures.^66^ However, the contact resistance can be effectively reduced through external modulation strategies, such as doping^66^ or interface polarization.^67^ These results suggest that the FeB_2_/SiC heterostructure provides a viable platform for tuning contact characteristics and holds promise for future applications in 2D Schottky electronic devices.

## 4 Conclusion

In summary, we have systematically investigated the structural stability, electronic properties, and interfacial contact characteristics of the two-dimensional FeB_2_/SiC metal–semiconductor heterostructure by means of first-principles calculations. The optimized heterostructure is found to be energetically stable, with a weak vdW interaction at the interface, which preserves the intrinsic electronic properties of the constituent FeB_2_ and SiC monolayers. Due to the larger work function of metallic FeB_2_ compared with that of the SiC monolayer, electrons transfer from the SiC layer to the FeB_2_ layer upon contact. This charge redistribution induces an internal electric field and results in downward band bending in the SiC layer, leading to the formation of an n-type Schottky contact at the FeB_2_/SiC interface. The calculated n-type Schottky barrier height is *Φ*_B,n_ = 0.70 eV, indicating a relatively low energy barrier for electron injection across the interface. Further analysis of the projected density of states indicates that metal-induced gap states (MIGS) are negligible at the FeB_2_/SiC interface, with no pronounced states emerging within the band gap of the SiC layer. Moreover, the FeB_2_/SiC heterostructure exhibits a small tunneling resistance of *ρ*_t_ = 1.40 × 10^−9^ Ω cm^2^, confirming the formation of a low-resistance metal–semiconductor contact. Such a reduced contact resistance is highly beneficial for efficient carrier injection and low-power device operation. Our findings provide fundamental insights into the interfacial physics of 2D metal–semiconductor heterostructures and highlight the FeB_2_/SiC heterostructure as a promising candidate for next-generation 2D electronic and optoelectronic devices.

## Conflicts of interest

There are no conflicts to declare.

## Data Availability

The data that support the findings of this study are available from the corresponding author upon reasonable request.
